# A systematic review and mixed-treatment comparison of dapagliflozin with existing anti-diabetes treatments for those with type 2 diabetes mellitus inadequately controlled by sulfonylurea monotherapy

**DOI:** 10.1186/1758-5996-6-73

**Published:** 2014-06-11

**Authors:** Michelle Orme, Peter Fenici, Isabelle Duprat Lomon, Gail Wygant, Rebecca Townsend, Marina Roudaut

**Affiliations:** 1 ICERA Consulting Ltd, 17 Redbridge Close, Swindon SN5 8ZL, UK; 2 Bristol-Myers Squibb, 3 rue Joseph Monier, Rueil-Malmaison 92500, France; 3 Bristol-Myers Squibb, 100 Nassau Park Blvd, Princeton, NJ 08543, USA; 4 Affiliation at time of study: AstraZeneca, Rue Egide van Ophemstraat 110, Brussels B-1180, Belgium

**Keywords:** Diabetes, Dapagliflozin, Mixed treatment comparison, Systematic review, Network meta-analysis

## Abstract

**Background:**

To compare the first-in-class sodium glucose co-transporter 2 (SGLT2) inhibitor, dapagliflozin, with existing type 2 diabetes mellitus (T2DM) treatment options available within the European Union (EU) for add-on therapy to sulfonylureas (SUs).

**Methods:**

A systematic review was conducted to identify randomised controlled trials (RCTs) in T2DM patients inadequately controlled by SU monotherapy. Direct meta-analysis, Bucher indirect comparisons and Bayesian network meta-analysis (NMA) were conducted on studies meeting predefined inclusion criteria. Sufficient data were available to assess three clinical endpoints at 24 (+/- 6) weeks follow-up: mean change in HbA1c from baseline, mean change in weight from baseline, and the proportion of patients experiencing at least one episode of hypoglycaemia. The effect of confounding baseline factors was explored through covariate analyses.

**Results:**

The search identified 1,901 unique citations, with 1,870 excluded based on title/abstract. From reviewing full-texts of the remaining 31 articles, 5 studies were considered eligible for analysis. All studies were comparable in terms of baseline characteristics, including: HbA1c, age and body mass index (BMI). In addition to dapagliflozin, sufficient data for meta-analysis was available for three dipeptidyl peptidase-4 (DPP-4) inhibitors and one glucagon-like peptide-1 (GLP-1) analogue. Based on fixed-effect NMA, all treatment classes resulted in statistically significant decreases in HbA1c at follow-up compared to placebo. Dapagliflozin treatment resulted in significantly decreased weight at follow-up compared to placebo (-1.54 kg; 95% CrI -2.16, -0.92), in contrast to treatment with GLP-1 analogues (-0.65 kg; 95% CrI -1.37, 0.07) and DPP-4 inhibitors (0.57 kg; 95% CrI 0.09, 1.06). The odds of hypoglycaemia were similar to placebo for dapagliflozin and DPP-4 inhibitor add-on treatment, but significantly greater than placebo for GLP-1 analogue add-on treatment (10.89; 95% CrI 4.24, 38.28). Assessment of NMA model heterogeneity was hindered by the small size of the network.

**Conclusions:**

Dapagliflozin, DPP-4 inhibitors and GLP-1 analogues, in combination with SU, all provided better short-term glycaemic control compared to SU monotherapy. Dapagliflozin was the only add-on therapy that had both a favourable weight and hypoglycaemia profile compared to the other classes of treatment evaluated.

## Background

Type 2 diabetes mellitus (T2DM) is becoming increasingly prevalent, primarily due to increases in obesity and physical inactivity. With this comes an associated increase in healthcare resource burden, including, but not limited to, the cost of anti-diabetes agents, hospital inpatient care and treatment of the complications of diabetes, such as retinopathy and amputation [1-6]. The primary treatment goal of diabetes management is to reduce glycaemic levels, lowering glycated haemoglobin (HbA1c) levels to below or around 7% [7-9], in order to most effectively reduce diabetes-related macro and microvascular complications [10-14]. More effective drug therapies, enabling better disease stabilisation, could therefore have a significant impact on reducing healthcare resources required to treat such complications. Guidelines for treatment management include lifestyle modification at diagnosis, followed by metformin monotherapy as a first-line pharmacological treatment in most cases [7,13,15]. However, for patients where metformin is contraindicated, sulfonylureas (SUs) can be used as an alternative first-line pharmacological intervention [13,15]. Contraindications to metformin are quite common among T2DM patients, with one retrospective study identifying as many as 60% metformin users as having at least 1 contraindication, of which heart failure and renal impairment were the most frequently present [16]. SUs exert their glucose-lowering effect by stimulating insulin secretion and are able to reduce HbA1c levels to a similar extent as metformin monotherapy [8].

However, the progressive nature of T2DM means that eventually disease control will be lost and treatment intensification or additional agents will be required. The classes of treatment licensed for use in combination with SUs within the EU include: dipeptidyl peptidase-4 (DPP-4) inhibitors, glucagon-like peptide-1 (GLP-1) analogues, thiazolidinediones (TZDs) and a new class of agent, sodium glucose co-transporter 2 (SGLT2) inhibitors. The selection of combination treatment options is complex due to the number of factors that must be considered, including any unintended consequences of treatment, such as hypoglycaemia and weight gain, as they can significantly impact patient adherence and quality of life [17-21].

Dapagliflozin, developed by Bristol-Myers Squibb and AstraZeneca, was the first SGLT2 inhibitor approved in the EU (April 2012) and demonstrates a novel and insulin-independent mechanism of action. SGLT2 inhibitors reduce glucose reabsorption from the proximal tubule of the kidney, leading to increased urinary glucose excretion, and an associated loss of calories [22,23]. Dapagliflozin is indicated in adults aged 18 years and older with T2DM both as a monotherapy and as add-on combination therapy with other glucose-lowering medicinal products including insulin, when diet and exercise do not provide adequate glycaemic control. Dapagliflozin has been studied in a comprehensive clinical trial programme, including placebo-controlled and head-to-head studies [24-32]. The efficacy and safety of dapagliflozin as an add-on to SUs has been studied in a placebo-controlled study [31], but data from head-to-head studies comparing dapagliflozin with other anti-diabetes treatments when used for this particular indication are not currently available.

To assess the relative effects of treatment comparators in the absence of head-to-head trials, the use of network meta-analysis (NMA) has been recommended [33-35]. Whereas standard meta-analyses evaluate the relative efficacy of just two treatments based on head-to-head trials only, NMA comprises an extension of these methods in which treatment effects are calculated for a network of treatments, using both direct and indirect evidence [36-38]. As for all analysis that pools trial data, the key assumption is that the trial design and the trial outcomes being assessed are sufficiently similar across the network [39,40].

The primary objective of this study was to estimate the relative effect of the novel agent dapagliflozin versus existing classes of anti-diabetes therapy on key outcomes of interest, including HbA1c, weight, systolic blood pressure, and hypoglycaemia, when used as add-on treatments to SUs for patients with T2DM inadequately controlled by SU monotherapy with diet and exercise.

## Methods

### Selection of studies

A study protocol was developed prior to the review to define the scope of both the systematic review and meta-analysis (Table 1). The inclusion criteria were designed to ensure that all included studies adhered to the European Medicines Agency (EMA) guidelines [41]. The dapagliflozin clinical trial [31] was used as the benchmark since the design concurs with the current EMA guidance on clinical development programmes for diabetes trials [41].

**Table 1:** Inclusion and exclusion criteria for SU add-on systematic review

**Inclusion criteria**	**Description**
Population	Adults (aged ≥18 years) with T2DM on a stable dose of a SU as monotherapy (for at least 8 weeks, at half the maximum dose or maximum tolerable dose) where SU alone, with diet and exercise, does not provide adequate glycaemic control.
Treatment pathway	Add-on pharmacological therapy after failure of SU monotherapy, where the first-line SU was initiated because metformin was considered inappropriate.
Interventions	Pharmacological therapies that would be added to a SU in clinical practice when SU monotherapy does not provide adequate glycaemic control.
Comparators	Active arms: Dual therapies of interest namely drugs/doses licensed in the EU, as a dual therapy in combination with a SU and as used in clinical practice [13].
Outcomes	To be included in the meta-analysis, studies needed to report at least 1 of the primary endpoints of interest at 24 +/- 6 weeks:
	• mean change in HbA1c from baseline
	• mean change in weight from baseline
	• mean change in systolic blood pressure from baseline
	• proportion (number) of patients experiencing at least 1 hypoglycaemia episode
	Interim results from longer-term studies were permitted provided that dose titration and a sufficient maintenance period were complete at follow-up.
Study design	• Prospective, RCTs.
	• If cross-over design then results reported prior to the cross-over period can be used in the meta-analysis.
	• Minimum follow-up of 18 weeks to be included in the meta-analysis (i.e. 24 weeks +/-6 weeks); an expansion of the study window to 24 +/- 8 weeks as a sensitivity analysis for borderline studies was permitted.
Publications	Full-text publications, except for abstracts published in 2012 (for results from recently completed trials), full-text available in English.
Exclusions	• Results from uncontrolled open label extensions of RCTs.
	• Studies of SU used as part of a triple therapy regimen.
	• Study populations with moderate to severe renal impairment.
	• Study arms using treatment dosing regimens that are not licensed in the EU.

EU, European Union; Hba1c, glycated haemoglobin; RCT, randomised-controlled trial; SU, sulfonylurea; T2DM, type 2 diabetes mellitus.

The target population was defined as adult (aged ≥18 years) T2DM patients, who despite receiving a stable dose of SU monotherapy for at least 8 weeks (at half the maximum dose or maximum tolerable dose), had inadequate glycaemic control (Table 1). The target population were required to receive a stable dose of SU monotherapy to ensure that the maximal effect of this medication was achieved and to ensure that HbA1c was stabilised at baseline [41]. Patients had received first-line SU therapy because metformin was considered inappropriate as per current clinical treatment guidelines [13]. Eligible studies were prospective randomised-controlled trials (RCTs), conducted in the target population, that evaluated therapies in combination with an SU as part of a dual therapy regimen. The interventions of interest were therapies (and specifically doses of the therapies) licensed in the EU for use in combination with a SU, and which comprise the treatment options at this stage of the treatment pathway. The specific comparators in this analysis therefore included: dapagliflozin (the first SGLT2 inhibitor), DPP-4 inhibitors, GLP-1 analogues and TZDs. Metformin and insulin were not included as comparators in this analysis since they are not relevant comparators at this stage of the treatment pathway in clinical practice [13]. Additionally, alpha-glucosidase inhibitors were not considered as they are not commonly used [42,43].

For inclusion in the meta-analysis, studies were required to report at least one of the primary endpoints of interest at 24 +/- 6 weeks. The outcomes of interest were pre-defined in the study protocol and selected based on clinical priorities: efficacy measures selected were mean change in HbA1c from baseline, mean change in weight from baseline, mean change in systolic blood pressure from baseline and proportion (number) of patients experiencing at least one episode of hypoglycaemia. The risk of hypoglycaemia, while being a safety outcome, was included as an outcome of interest because it is of primary importance to the management of T2DM. Current EMA guidelines state that therapeutic confirmatory studies should typically be of 6 months duration [41]. Since not all studies report endpoints at 24 weeks, a study window of 24 weeks +/- 6 weeks was selected; all included studies would therefore have a minimum of an 18 week follow-up period, corresponding to the EMA guidelines for minimum recommended maintenance and titration periods (16 weeks plus 2 weeks, respectively) [41]. The pre-defined study protocol also permitted an expansion of the study window to 24 +/- 8 weeks as a sensitivity analysis for borderline studies.

A number of study exclusion criteria were also applied (Table 1). These included results from open label extensions of RCTs and studies of SU as part of a triple therapy regimen.

### Systematic review methodology

A systematic review was conducted, using a structured search (Additional file 1) via the OVID platform of the following electronic databases: CENTRAL (March 2013), Medline and Medline in Process (1946 to 9th April 2013), and Embase (1980 to 2013 week 14) [44]. The search strategy employed was set up to be sensitive, to ensure that all relevant publications would be retrieved by the search, although this strategy would also retrieve a high proportion of irrelevant abstracts which would subsequently need to be excluded during the screening process. In addition, the 2012 and 2013 (where available) conference proceedings from the following organisations were searched: American College of Cardiology (ACC), American Diabetes Association (ADA), American Heart Association (AHA), European Association for the Study of Diabetes (EASD), The Obesity Society and The International Diabetes Federation. The clinical trials registry (ClinicalTrials.gov) was hand-searched for unpublished trials. Citations were included in the systematic review if they met the pre-defined inclusion criteria (Table 1). The initial screening for relevant studies was based on the citation abstract and title. For the second stage, the full-text publication was retrieved and the citations were re-screened to ensure their eligibility for inclusion in the systematic review. Disputes as to eligibility were referred to a third reviewer (PF). A quality assessment of the included studies was conducted using the Cochrane Collaboration’s tool for assessing risk of bias [45].

### Meta-analysis

All meta-analyses were conducted on a modified intent-to-treat population, which is defined as the set of patients who were randomised and received at least 1 dose of study medication. For some studies the efficacy analysis set was further restricted to those with baseline and ≥1 follow-up efficacy result. Due to the limited number of studies available, study arms were pooled by drug class to improve accuracy by increasing the amount of data available for each class-level comparison. The pooled summary measure for continuous endpoints is weighted mean difference (WMD) and for binomial outcomes is the odds ratio (OR). ORs were utilised in order to facilitate indirect comparisons using the Bucher method and the logit (log odds) model within the NMA [46,47]. For continuous endpoints, both the mean and standard error were required for the meta-analysis. If the standard error was not reported then this was calculated from the confidence interval (CI) or the standard deviation (SD), or imputed from the observed standard errors using the method described in the Cochrane Handbook for Systematic Reviews of Interventions [48].

### Direct meta-analysis and Bucher indirect comparisons

Fixed and random-effects direct meta-analyses were conducted in Stata IC version 12.1 using the metan package SJ9_2: sbe24_3 [49,50]. The random-effects model uses the method of DerSimonian & Laird, with the estimate of heterogeneity being taken from the fixed-effect Mantel-Haenszel or inverse variance model. The inverse variance approach calculates study weight based on the assumption that variance is indirectly proportional to the study importance, whereas the Mantel-Haenszel approach uses a similar methodology whilst assuming a fixed treatment effect. In most instances, the between-study heterogeneity based on the I^2^ statistic from the direct random-effects meta-analysis could not be assessed due to the limited scope of the analysis.

Simple indirect comparisons were made using the Bucher method, in order to provide an assessment of consistency for the NMA [46,51].

### Network meta-analysis

In an NMA, treatment effects are calculated for all treatments using all available evidence in one simultaneous analysis [36-38]. NMA methods build on the principles of indirect comparisons and preserve the randomised comparisons within each trial [46,51]. The NMA methodology was based on the National Institute for Health and Care Excellence (NICE) Decision Support Unit recommendations for random and fixed effect Bayesian network meta-analysis: for mean change in HbA1c from baseline and mean change in weight from baseline, a normal NMA model with identity link was used; for count of patients with hypoglycaemia, a binomial model with logit link was used [34].

All models were fitted to the data via Bayesian Markov Chain Monte Carlo methods (specifically Gibbs sampling) using WinBUGS [52]. The WinBUGS models were run for a minimum of 100,000 iterations to ensure model convergence. An estimate of how well the values predicted by the model fitted the observed dataset was provided by the mean residual deviances (total residual deviance divided by number of datapoints) as well as the deviance information criteria (DIC) output by WinBUGs [34]. The model with the lowest DIC output by WinBUGs was deemed to best predict a replicate dataset of the same structure of that observed, provided differences were more than 5 [53,54].

Both random-effects and fixed-effect models were tested. Random-effects NMA allows the true treatment effect (eg. OR between two treatments) to vary between studies due to heterogeneity. In these random-effects models, a uniform (uninformative) prior was used for the between-studies SD (as per Hasselblad [55] and Gelman [56]). However, due to the limited number of studies involved in this analysis, fixed-effect models were preferred in all instances.

The absolute change for each treatment was calculated using the relative effects and the baseline risk/absolute change for the reference arm [57]. The reference treatment was the arm with the most data available, ie. placebo-control.

Previous meta-analysis have shown that there is a correlation between baseline HbA1c and change in HbA1c over follow-up [58]. Furthermore it is known that glycaemic control is harder to achieve in overweight or obese patients [59]. To account for the potential differences in baseline values across RCTs, and given the potential effect modification that can be attributed to baseline HbA1c and baseline weight, covariate analyses using baseline values were performed for both HbA1c and weight primary endpoints using the methodology recommended in Evidence Synthesis, Technical Support Document 3 [60].

## Results

### Systematic review search results

**Figure 1:**
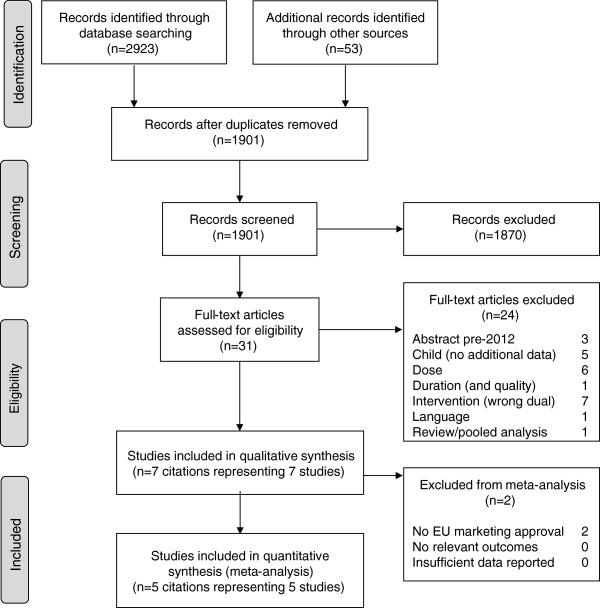
Flow diagram of study selection (systematic review).

The OVID database search retrieved 2,923 citations of which 1,848 were unique. In addition, 52 abstracts were retrieved from the conference proceedings search and 1 unpublished study was identified from the clinical trial registry. After reviewing the title/abstract, 1,870 citations were excluded, with a further 24 excluded following full-text review (Additional file 2). Seven citations (representing 7 different studies) were eligible for inclusion in the systematic review, with 5 studies fulfilling the eligibility criteria for inclusion in the meta-analysis (Figure 1, Table 2).

**Table 2:** Studies identified by the systematic review and eligible for inclusion in the NMA

**Author**	**Study setting**	**Duration, weeks**	**N, randomised**	**Class**	**Study arms**^**a**^**, treatment and dose (n)**	**SU background during trial**	**Pre-trial SU use**
Buse [61]	US	30	377	GLP-1	1) exenatide 5 μg BID (125);	Unchanged from baseline SU^b,c^	At least the maximally effective dose of a SU^c^ as monotherapy for ≥3 months before screening
					2) exenatide 10 μg BID (129);		
					3) placebo BID (123)		
Garber [62]	US, Sweden, Finland, Argentina, Lithuania	24	515	DPP-4	1) vildagliptin 50 mg QD (170);	Glimepiride 4 mg QD, reduced to 2 mg QD if hypoglycaemia occurred	≥7.5 mg glyburide or glipizide QD, or ≥2 mg glimepiride or equivalent, treated for >3 months with stable dose for ≥4 weeks before screening, switched to glimepiride 4 mg QD for 4 weeks prior to baseline
					*2) vildagliptin 100 mg QD* (169);		
					3) placebo QD (176)		
Hermansen [63]	Multi-country	24	212^d^	DPP-4	1) sitagliptin 100 mg QD (106);	Stable dose of glimepiride (4 mg – 8 mg QD)	A stable dose of glimepiride 4 – 8 mg QD for ≥10 weeks + 2 week run-in
					2) placebo QD (106)		
Lewin [64]	US, Argentina, India, Japan, Hungary, Poland, Russia	18	245	DPP-4	1) linagliptin 5 mg QD (161);	Unchanged from baseline SU^b,c^	Stable SU^c^ dose of ≥ half the maximum dose for 10 weeks (or documented maximum tolerated dose for ≥12 weeks) + 2 week run-in
					2) placebo QD (84)		
Strojek [31]	Czech Republic, Hungary, Poland, Ukraine, Republic of Korea, Philippines, Thailand	24	597	SGLT2	1) *dapagliflozin 2.5 mg QD (154);*	Glimepiride 4 mg QD, reduced to 2 mg QD or discontinued if hypoglycaemia occurred	Stable SU^c^ dose of ≥ half the maximum dose for 8 weeks. Continued on or switched to glimepiride 4 mg/day during an 8 week run-in
					2) *dapagliflozin 5 mg QD (145);*		
					3) dapagliflozin 10 mg QD (151);		
					4) placebo QD (146)		

^a^Study arms in italics were not included in the meta-analysis (not EU licensed dose); ^b^Down-titration allowed in response to hypoglycaemia; ^c^SU not specified in the publication; ^d^Strata 1 (glimepiride only subgroup) n = 212, full trial population n = 441; BID, twice daily; DPP-4, dipeptidyl peptidase-4 inhibitors*;* GLP-1, glucagon-like peptide-1 analogues; NMA, network meta-analysis; QD, once daily; SGLT2, sodium glucose co-transporter 2 inhibitors; SU, sulfonylurea.

### Summary of included and excluded studies

**Figure 2:**
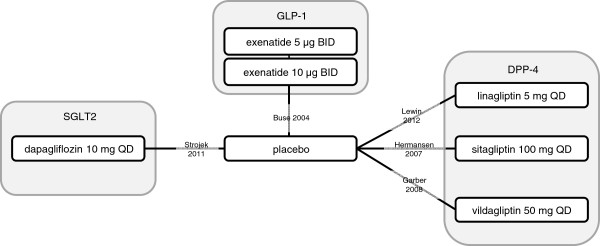
Network diagram for studies meeting criteria for inclusion in the meta-analysis.

Three classes of anti-diabetes treatment were covered by the 5 studies fulfilling the inclusion criteria (Figure 2): DPP-4 inhibitors (3 studies), GLP-1 analogues (1 study) and SGLT2 inhibitors (1 study). All studies identified were placebo-controlled. Study duration ranged from 18 weeks to 30 weeks, including 3 studies that reported endpoints at the 24 week benchmark (Table 2) [31,61-64]. Overall the included studies were comparable in terms of HbA1c, age and body mass index (BMI) patient entry criteria, and the baseline characteristics were similar across the studies (Additional file 3). The quality assessment of the included studies indicated a low risk of bias overall (Additional file 4).

Of the 24 studies excluded following review of the full-text, 6 studies were excluded based on the dose of SU received at randomisation (Additional file 2). A stable dose of SU was critical not only to ensure that SU treatment had reached maximum efficacy but also to avoid potentially confounding differences in HbA1c at baseline. An additional 7 studies were excluded because they did not evaluate a dual therapy comparison of interest (Additional file 2).

From the studies eligible for inclusion in the meta-analysis, sufficient data were reported for 3 of the key outcomes at the desired follow-up: change in HbA1c, change in weight and number of patients reporting hypoglycaemia. Only 2 studies reported mean change in systolic blood pressure and neither reported the corresponding standard errors. As a result this outcome was not analysed due to insufficient data.

### Direct meta-analysis

**Figure 3:**
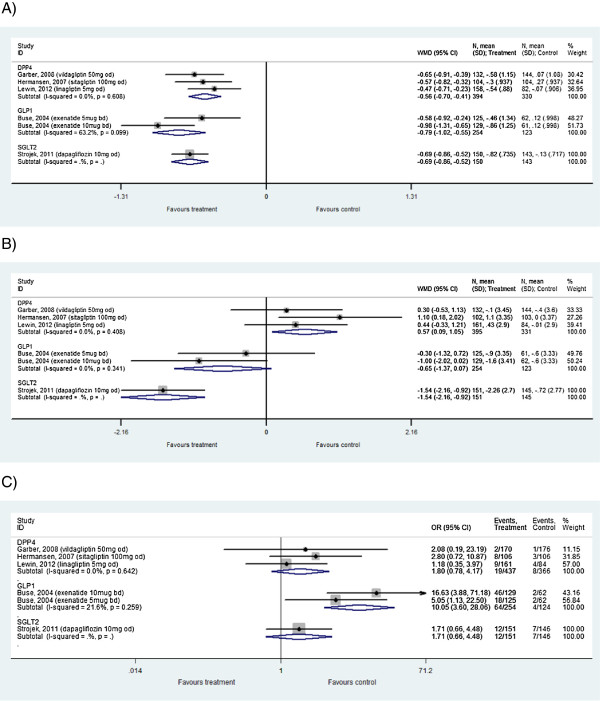
**Direct meta-analysis forest plots versus placebo-control. A)** HbA1c weighted mean difference, **B)** weight (kg) weighted mean difference, **C)** hypoglycaemia odds ratio; CI, confidence interval; DPP-4, dipeptidyl peptidase-4 inhibitors; GLP-1, glucagon-like peptide-1 analogues; N, number of patients; OR, odds ratio; SD, standard deviation; SGLT2, sodium glucose co-transporter 2 inhibitors; WMD, weighted mean difference.

Based on the fixed-effect direct meta-analysis, all classes of anti-diabetes treatments resulted in a statistically significant greater decrease in HbA1c at follow-up compared to placebo (p < 0.01) (Table 3). Dapagliflozin treatment resulted in a statistically significant larger decrease in weight at follow-up compared to placebo (-1.54 kg [95% CrI: -2.16, -0.92]; p < 0.01), whereas DPP-4 inhibitor therapy resulted in a statistically significant increase in weight at follow-up compared to placebo (0.57 kg [95% CrI: 0.09, 1.06]; p < 0.02). GLP-1 analogue treatment did not lead to a statistically significant change in weight but it did result in a statistically significant higher odds of hypoglycaemia at follow-up compared to placebo (10.89 [95% CrI: 4.24, 38.28]; p < 0.01). In contrast, the odds of hypoglycaemia for dapagliflozin and DPP-4 inhibitors were not significantly different to placebo (p > 0.05) (Table 3, Figure 3).

**Table 3:** Results of the fixed-effect direct, Bucher indirect and NMA comparisons

**Comparison**	**HbA1c**		**Weight (kg)**		**Hypoglycaemia**	
	**NMA (95% CrI)**	**Direct/Bucher indirect (95% CI)**	**NMA (95% CrI)**	**Direct/Bucher indirect (95% CI)**	**NMA (95% CrI)**	**Direct/Bucher indirect (95% CI)**
	*WMD v placebo*	*Direct comparison*	*WMD v placebo*	*Direct comparison*	*OR v placebo*	*Direct comparison*
DPP-4 v placebo	-0.56 (-0.70, -0.41)†	-0.56 (-0.70, -0.41)*	0.57 (0.09, 1.06)†	0.57 (0.09, 1.05)*	1.87 (0.82, 4.72)	1.8 (0.78, 4.17)
GLP-1 v placebo	-0.80 (-1.04, -0.56)†	-0.79 (-1.02, -0.55)*	-0.65 (-1.37, 0.07)	-0.65 (-1.37, 0.07)	10.89 (4.24, 38.28)†	10.05 (3.60, 28.06)*
SGLT2 v placebo	-0.69 (-0.86, -0.52)†	-0.69 (-0.86, -0.52)*	-1.54 (-2.16, -0.92)†	-1.54 (-2.16, -0.92)*	1.75 (0.67, 4.89)	1.71 (0.66, 4.48)
	*WMD head-to-head*	*Bucher indirect comparison*	*WMD head-to-head*	*Bucher indirect comparison*	*OR head-to-head*	*Bucher indirect comparison*
GLP-1 v DPP-4	-0.24 (-0.52, 0.04)	-0.23 (-0.50, 0.04)	-1.23 (-2.09, -0.36)†	-1.22 (-2.09, -0.35)*	5.89 (1.56, 26.06)†	5.58 (1.49, 20.99)*
SGLT2 v DPP-4	-0.13 (-0.35, 0.09)	-0.13 (-0.35, 0.09)	-2.11 (-2.90, -1.33)†	-2.11(-2.90, -1.33)*	0.94 (0.25, 3.50)	0.95 (0.27, 3.37)
SGLT2 v GLP-1	0.11 (-0.18, 0.40)	0.10 (-0.19, 0.39)	-0.89 (-1.84, 0.07)	-0.89 (-1.84, 0.06)	0.16 (0.03, 0.65)†	0.17 (0.04, 0.69)*
	*WMD v baseline*		*WMD v baseline*		*Probability of hypoglycaemia*	
placebo	0.05 (-0.10, 0.20)	N/A	-0.48 (-0.78, -0.18)†	N/A	3.99% (2.30%, 6.85%)	N/A
DPP-4	-0.51 (-0.72, -0.30)†	N/A	0.09 (-0.47, 0.66)	N/A	7.23% (2.75%, 18.67%)	N/A
GLP-1	-0.75 (-1.03, -0.47)†	N/A	-1.13 (-1.91, -0.35)†	N/A	31.38% (13.00%, 64.10%)	N/A
SGLT2	-0.64 (-0.87, -0.42)†	N/A	-2.02 (-2.71, -1.33)†	N/A	6.81% (2.33%, 19.04%)	N/A
	*Model parameter results*					
DIC (WinBUGs)	-10.36	N/A	13.07	N/A	72.301	N/A
Average resdev	1.37	N/A	1.14	N/A	2.25	N/A

*Statistically significant result (p < 0.05); †statistically significant based on 95% CrI; CI, confidence interval; CrI, credible interval; DIC, deviance information criterion; DPP-4, dipeptidyl peptidase-4 inhibitors; GLP-1, glucagon-like peptide-1 analogues; OR, odds ratio; NMA, network meta-analysis; resdev, residual deviance; SGLT2, sodium glucose co-transporter 2 inhibitors; WMD, weighted mean difference.

Between-study heterogeneity, based on the I^2^ statistic from the direct random-effects meta-analysis, was assessed for DPP-4 inhibitors only, since no other class of therapy had more than one trial. No significant heterogeneity between the 3 DPP-4 inhibitor trials was observed for any of the outcomes: HbA1c I^2^ = 0%, p = 0.61; weight I^2^ = 0%, p = 0.41; hypoglycaemic events I^2^ = 0%, p = 0.64 (Figure 3).

### Bucher indirect comparisons

Pairwise Bucher indirect comparisons revealed a statistically significant difference in the weighted mean difference in weight between dapagliflozin and DPP-4 inhibitors (-2.11 kg [95% CI: -2.90, -1.33; p < 0.01]) and between GLP-1 analogues and DPP-4 inhibitors (-1.22 kg [95% CI: -2.09, -0.35; p = 0.01]). It also demonstrated that the odds of hypoglycaemia with dapagliflozin treatment or DPP-4 inhibitor treatment are significantly lower than GLP-1 analogue treatment (0.17 [95% CI: 0.04, 0.69; p = 0.01] and 0.18 [95% CI: 0.05, 0.67; p = 0.01] respectively). All other Bucher indirect comparisons showed no statistically significant differences in outcomes between treatments (Table 3).

### NMA

**Figure 4:**
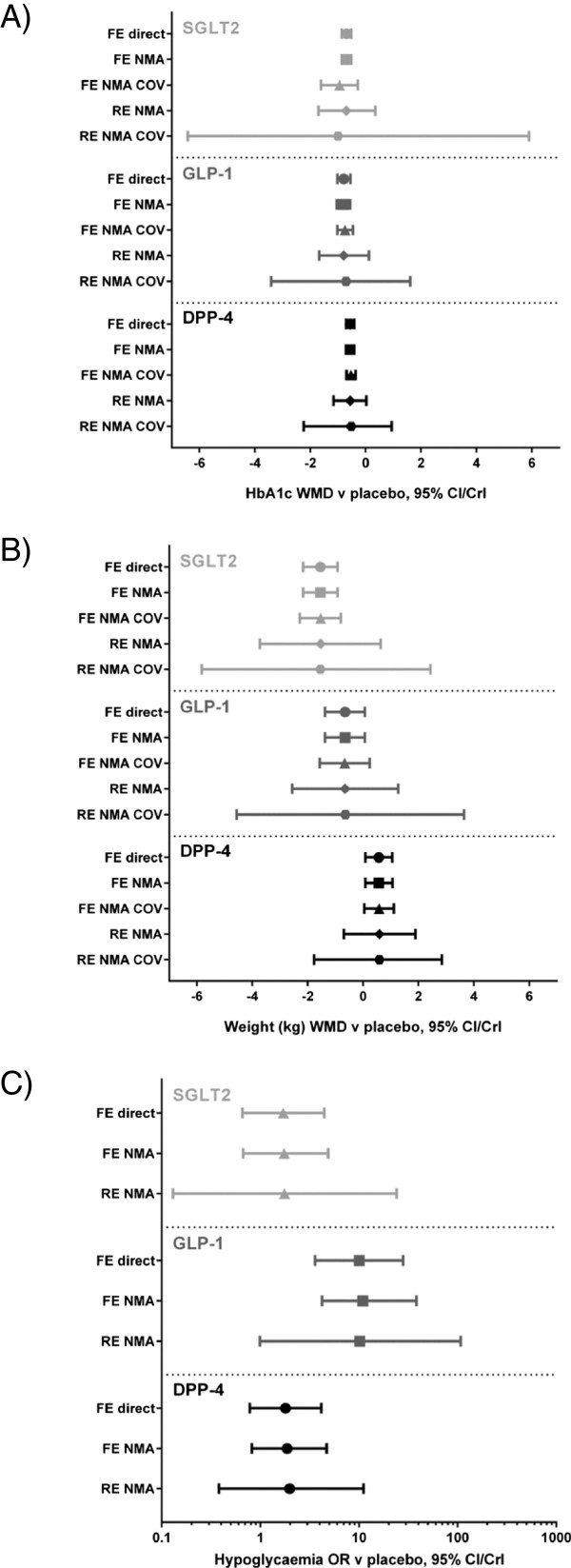
**Sensitivity analysis across different meta-analysis models. A)** HbA1c, weighted mean difference, **B)** weight (kg), weighted mean difference, **C)** hypoglycaemia odds ratio; DPP-4, dipeptidyl peptidase-4 inhibitors; direct, direct meta-analysis; FE, fixed-effect; GLP-1, glucagon-like peptide-1 analogues; NMA, network meta-analysis; OR, odds ratio; RE, random-effects; SGLT2, sodium glucose co-transporter 2 inhibitors; WMD, weighted mean difference.

For the HbA1c data, the random-effects model without covariates provided the lowest DIC value. However, due to the low number of studies in the network, the random-effects model overestimates the uncertainty, which is evident from the high between study SD and large credible intervals (CrI) which includes an unrealistic range of values (Figure 4). The most reliable estimates in terms of CrI were therefore provided by the fixed-effect NMA, which demonstrated that all 3 classes of treatment resulted in statistically significant larger decrease in HbA1c at follow-up compared to placebo (based on the 95% CrI) (Table 3). The coefficients estimating the impact of baseline HbA1c on treatment effects were not significant for either the fixed or random-effects models (Figure 4). It was noted, however, that the inclusion of the baseline HbA1c covariate had a larger impact on dapagliflozin than the DPP-4 inhibitor and GLP-1 analogue classes. This may be explained by the observation that for both DPP-4 inhibitors and GLP-1 analogue study arms, baseline HbA1c was close to the average baseline, while the baseline HbA1c for the dapagliflozin trial was lower than the study arm average.

The fixed-effect model provided the best overall fit for the change in weight data (Table 3). As with the HbA1c outcome, both random-effects models had high between study SD and large CrI, indicating substantial uncertainty in the estimates from these models (Figure 4). Based on the fixed-effect NMA, dapagliflozin treatment resulted in a significantly larger decrease in weight at follow-up compared to placebo (-1.54 kg [95% CrI: -2.16, -0.92]), which is in contrast to treatment with GLP-1 analogues (-0.65 kg [95% CrI: -1.37, 0.07]) and DPP-4 inhibitors (0.57 kg [95% CrI: 0.09, 1.06]). The coefficients estimating the impact of baseline weight on treatment effects were not significant for either the fixed or random-effects covariate models.

Two models were evaluated as part of the NMA for the hypoglycaemia endpoint; fixed-effect and random-effects models. The random-effects model provided the best overall fit for the hypoglycaemia data, but both models produced large CrI (Figure 4), and the between-study SD was high. Given the lack of studies, the fixed-effect NMA provides the most robust estimates (Table 3), and indicates that the odds of hypoglycaemia for placebo were similar to dapagliflozin and DPP-4 inhibitor add-on treatment, but significantly greater than placebo for GLP-1 analogue add-on treatment (10.89 [95% CrI: 4.24, 38.28]).

Results of pairwise comparisons estimated from the NMA were comparable to the Bucher indirect estimates (Table 3). No statistically significant differences in weighted mean difference for HbA1c were seen between drug treatments. Both dapagliflozin and GLP-1 analogues resulted in a larger decrease in weight compared to DPP-4 inhibitors, with the weighted mean difference being larger for dapagliflozin vs DPP-4 inhibitors than for GLP-1 analogues vs DPP-4 inhibitors (-2.11 kg [95% CrI: -2.90, -1.33] and -1.23 kg [95% CrI: -2.09, -0.36] respectively). Although dapagliflozin resulted in a larger decrease in weight compared to GLP-1 analogues, this was not statistically significant. Both dapagliflozin and the DPP-4 inhibitors demonstrated statistically significant lower odds of hypoglycaemia compared to GLP-1 analogues (0.16 [95% CrI: 0.03, 0.65] and 0.17 [95% CrI: 0.04, 0.64] respectively). No other statistically significant differences between treatments were seen.

## Discussion

This study was conceived to estimate the relative effect of the SGLT2 inhibitor dapagliflozin, compared with existing anti-diabetes therapies when used as add-on treatments to SUs for those with T2DM inadequately controlled by SU monotherapy, thereby permitting more informed treatment choices in clinical practice.

Of the existing anti-diabetes treatments licensed for such use in the EU, sufficient data was available to compare dapagliflozin with 2 other classes of agent in the current meta-analysis: DPP-4 inhibitors and GLP-1 analogues. Canagliflozin, an alternative SGLT2 inhibitor available in the EU, was not included in this analysis as the objective was not to compare the SGLT2 class with other available treatments, but to compare dapagliflozin. Furthermore, no relevant studies have been identified for canagliflozin that would have met the inclusion criteria for this study. Three outcomes were evaluated based on clinical priorities and data availability, including the risk of hypoglycaemia. Specific safety outcomes other than hypoglycaemia were not included in the current analysis. The relative rates of key adverse events observed in placebo-controlled trials of dapagliflozin have previously been meta-analysed using data from across the dapagliflozin clinical trial program [65,66]. Safety profiles of DPP-4 inhibitors and GLP-1 analogues have also been extensively compared and reviewed [67-71].

Results highlighted that all 3 classes of anti-diabetes treatments used as add-on therapy to SU monotherapy provided better short-term glycaemic control than an SU used alone, and may suggest that no single class of treatment was able to significantly reduce HbA1c to a greater extent than any other. It should however be noted that this analysis is underpowered. Interestingly, the analysis demonstrated that there were differences between classes of treatment in terms of impact on weight and the incidence of hypoglycaemic events. The definition of hypoglycaemia was broadly similar between studies (requiring symptomatic confirmation) with the notable exception of one of the three DPP-4 inhibitor studies [64]. Despite this difference there was not considered to be any significant bias; the most likely result of the difference in definition would be an underestimate of hypoglycaemia compared to the other studies, but the percentage of hypoglycaemic events reported in the placebo arm of this study was comparable to the dapagliflozin study, suggesting that this was not the case [31,64]. Of note was the increased incidence of hypoglycaemia in the GLP-1 analogue class of treatment compared to dapagliflozin and DPP-4 inhibitors. The GLP-1 analogue trial publication noted that the increase in hypoglycaemia observed in this study was likely caused by background susceptibility to hypoglycaemia often observed in SU treated patients coupled with lower ambient glycaemia [61]. It should be noted however that only a single GLP-1 analogue study was included in the meta-analyses and that the discontinuation rate was elevated compared to the other included studies.

Weight gain and hypoglycaemia are commonly documented side effects for SU monotherapy [12,13,72,73] and from a patient-centred perspective, the impact that weight gain and hypoglycaemia risk can have on patient quality of life and compliance is significant [17-20]. The patient population presented in this analysis were treated with half the maximum or the maximum tolerated dose of an SU, and would therefore be at increased risk of such side effects should the SU dose be raised in order to increase glycaemic control. Dapagliflozin was the only add-on therapy that had both a favourable weight and hypoglycaemia profile compared to the other classes of treatment evaluated.

### Strengths and limitations of the analysis

A general limitation of meta-analysis is the underlying assumption that trials and outcomes are sufficiently similar to allow for data to be pooled. In order to ensure that this assumption held in this analysis, the systematic review was designed to be highly specific with strict inclusion criteria. All exclusions were made based on the pre-defined criteria, and primarily designed to ensure that the analysis remained focused on a specific target population and clinically relevant comparators. This focus to the review and NMA also makes the results meaningful to clinical decision making and appropriate for cost-effectiveness analysis [74].

There will always be differences between studies, and some degree of heterogeneity in the analysis may provide a measure of uncertainty in effects due to differences in clinical practice. One potential source of heterogeneity is differences in follow-up times across studies. A 24 week timepoint was chosen since most registration studies are of this duration. The follow-up was allowed to vary by 24 +/- 6 weeks to allow for a pooled analysis of short-term endpoints, without impacting on weight and hypoglycaemia endpoints, which would accumulate over time. The impact of differences in SU background treatment was not considered to be a major limitation in this analysis since trial arms were balanced at baseline in terms of SU treatment and the SU dose was stable during the trial. Of the five studies eligible for inclusion, three studies used glimepiride as the background SU during follow-up, and while two studies did not specify the SUs used as background treatment, prior SU treatment duration was at least 8 weeks pre-enrolment for all studies (Table 2).

The limited availability of studies has hampered our assessment of heterogeneity by limiting the scope of the sensitivity analysis that can be conducted. As a result, there are insufficient studies with which to estimate the between-study variance with precision; therefore the CrI in the random-effects models may reflect uncertainty due to a lack of data rather than the true variance in treatment effects. Based on the size of network on which the analyses were performed therefore, the fixed-effect analyses are viewed to be the most robust estimates of treatment effects.

Results obtained from the NMA were consistent with results from the direct meta-analysis and Bucher indirect comparisons. This is due to the ‘star’ shaped network whereby all studies are connected via the placebo-control. In addition, the analyses presented here have been performed on a small number of studies. One advantage of NMA over the Bucher method is that it uses all the available placebo data in one analysis, while the Bucher method incorporates only some of the placebo data in each pairwise comparison. Inclusion of all the available data for NMA typically increases the power of the analysis. However this was not the case in this analysis given the small number of studies.

## Conclusions

Dapagliflozin, the first-in-class SGLT2 inhibitor was compared with 2 classes of anti-diabetes treatments licensed in the EU for use as add-on therapy to SUs for patients with T2DM in the current NMAs. All 3 classes of treatment provided better short-term glycaemic control when used in combination with an SU compared to SU monotherapy, with no significant differences between classes. However, NMA revealed that there were differences between dapagliflozin and the other classes of treatment in terms of impact on weight (dapagliflozin compared to DPP-4 inhibitors) and incidence of hypoglycaemia (dapagliflozin compared to GLP-1 analogues). Careful consideration and comparison of drug class risk-benefits should be made when selecting appropriate add-on drug combinations for the treatment of T2DM.

## Abbreviations

ACC: American college of cardiology; ADA: American diabetes association; AHA: American heart association; BID: Twice daily; BMI: Body mass index; CI: Confidence intervals; CrI: Credible interval; DIC: Deviance information criteria; DPP-4: Dipeptidyl peptidase-4; EASD: European association for the study of diabetes; EMA: European medicines agency; EU: European union; FE: Fixed-effect; GLP-1: Glucagon-like peptide-1; HbA1c: Glycated haemoglobin; NICE: National institute for health and care excellence; NMA: Network meta-analysis; OR: Odds ratio; QD: Once daily; RCTs: Randomised controlled trials; RE: Random-effects; SD: Standard deviation; SGLT2: Sodium glucose co-transporter 2; SU: Sulfonylureas; T2DM: Type 2 diabetes mellitus; TZDs: Thiazolidinediones; WMD: Weighted mean difference.

## Competing interests

This study was funded by Bristol-Myers Squibb Rueil-Malmaison, France and AstraZeneca, Brussels, Belgium. MO is a paid consultant of Bristol-Myers Squibb. PF, IDL, GW and MR are employees of Bristol-Myers Squibb. IDL and GW are also shareholders of Bristol-Myers Squibb. RT was an employee of AstraZeneca throughout the duration of the study.

## Authors’ contributions

All authors meet the Uniform Requirements for Manuscripts Submitted to Biomedical Journals criteria for authorship: all have made substantial contributions to the conception and design, acquisition of data or analysis and interpretation of the data, and the manuscript has been reviewed thoroughly and approved by all of the authors.

## Supplementary Material

Additional file 1 Database search strategy: Embase 1980 to 2013 week 13. Click here for file

Additional file 2 Summary of citations excluded after full-text review. Click here for file

Additional file 3 Key baseline characteristics for studies included in qualitative systematic review. Click here for file

Additional file 4 Risk of bias summary graph. Click here for file
