# Supplementary material for: A systematic review and mixed-treatment comparison of dapagliflozin with existing anti-diabetes treatments for those with type 2 diabetes mellitus inadequately controlled by sulfonylurea monotherapy
Source: Diabetol Metab Syndr. 2014 Jun 11;6:73. doi: 10.1186/1758-5996-6-73 (PMC4085736; doi:10.1186/1758-5996-6-73)
Supplement: Additional file 1 — Database search strategy: Embase 1980 to 2013 week 13. [file 1758-5996-6-73-S1.pdf]

Table A.1. Database search strategy: Embase <1980 to 2013 week 13>

Search term number	Search term
1	sulfonylurea/
2	sulfonylurea.mp.
3	sulphonylurea.mp.
4	glyburide.mp.
5	glibenclamide/
6	glibenclamide.mp.
7	gliclazide/
8	gliclazide.mp.
9	exp glimepiride/
10	glimepiride.mp.
11	glipizide/
12	glipizide.mp.
13	gliquidone/
14	gliquidone.mp.
15	glyclopyramide.mp.
16	tolbutamide/
17	tolbutamide.mp.
18	tolbutamide/
19	tolbutamide.mp.
20	or/1-19
21	exp drug combinations/
22	(drug therap$ or drug combination$).mp.
23	((combination$ or oral or multiple) adj (therap$ or agent$ or drug$ or treatment$)).mp.
24	monotherap$.mp.
25	or/21-24
26	20 and 25
27	exp non insulin dependent diabetes mellitus/
28	(MODY or NIDDM or T2DM).mp.
29	(non insulin$ depend$ or noninsulin$ depend$ or noninsulin?depend$ or non insulin?depend$).mp.
30	((typ$ 2 or typ$ II) adj diabet$).mp.
31	((keto?resist$ or non?keto$) adj diabet$).mp.
32	((late or adult$ or matur$ or slow or stabl$) adj diabet$).mp.
33	or/27-32
34	randomized controlled trial/
35	controlled clinical trial/
36	randomi?ed.ab.
37	placebo.ab.
38	randomly.ab.
39	trial.ab.
40	or/34-39
41	animal/
42	human/
43	41 not (41 and 42)
44	40 not 43
45	26 and 33 and 44
	TOTAL CITATIONS RETRIEVED: 1,570

ab, abstract; mp, multipurpose; sh, subject heading; ti, title
