# Supplementary material for: A systematic review and mixed-treatment comparison of dapagliflozin with existing anti-diabetes treatments for those with type 2 diabetes mellitus inadequately controlled by sulfonylurea monotherapy
Source: Diabetol Metab Syndr. 2014 Jun 11;6:73. doi: 10.1186/1758-5996-6-73 (PMC4085736; doi:10.1186/1758-5996-6-73)
Supplement: Additional file 2 — Summary of citations excluded after full-text review. [file 1758-5996-6-73-S2.pdf]

Table A.2. Summary of citations excluded after full-text review

Citation Details	Exclusion reason
Blonde, 2006. Obesity & Metabolism 8: 436-447	Child citation with no additional data (linked to Buse, 2004).
Hardy, 2011. Diabetologia 54: S344-S345.	Abstract only (pre-2012 cut-off).
Hardy, 2012. Diabetes 61: A252.	Review /pooled analysis.
Inagaki, 2012. European Association for the Study of Diabetes: Abstract 847	Not a dual therapy comparison of interest (SU or α-glucosidase inhibitor (A-GI)+metformin v (SU or A-GI)+linagliptin).
Kadowaki, 2011. Journal of Diabetes Investigation 2: 210-217.	Not a dual therapy comparison of interest (triple).
Kaku, 2010. Diabetes, Obesity & Metabolism 12: 341-347.	SU dose at randomisation (not stable dose for minimum of 4 weeks /not half maximum dose); Liraglutide dose lower than EU dose (0.6 mg, 0.9 mg).
Kikuchi, 2010. Diabetes Research and Clinical Practice 89: 216-223.	SU dose at randomisation (not stable dose for minimum of 4 weeks /not half maximum dose).
Kipnes, 2001. American Journal of Medicine 111: 10-17.	Duration of 16 weeks (less than recommended minimum 18 week follow-up period), SU run-in of 4 weeks, background includes (non-standard) individualised weight management but lack of data on weight measurements pre and during trial (baseline not reported, SE in change not reported), entry criteria of HbA1c of 8%< (very poor control), SEs not reported for primary endpoints.
Lester, 2005. International Journal of Clinical Practice 59: 134-142.	Pooled post-hoc analysis of four RCTs. Not a dual therapy comparison of interest (met+su v pio + su or su + met v pio + met).
Lu, 2011. Chinese Journal of Internal Medicine 50: 826-830	Language non-English full text (Chinese).
Marre, 2009. Diabetic Medicine 26: 268-278.	SU dose at randomisation (not stable dose for minimum of 4 weeks). Entry criteria were any oral glucose lowering therapy (including combinations). Switch to SU involved SU titration and maintenance period of only 2-4 weeks pre-randomisation.
Nauck, 2009. Postgraduate Medicine 121: 5-15.	Child citation with no additional data (linked to Marre, 2009).
Pan, 2002. Chung-Hua Nei Ko Tsa Chih Chinese Journal of Internal Medicine 41: 388-392.	Not a dual therapy comparison of interest (mixed metformin/ SU).
Parikh, 2012. Diabetologia 55: S305.	Child citation with no additional data (linked to Strojek, 2011).
Rajagopalan, 2005. Current Medical Research & Opinion 21: 163-172.	Pooled post-hoc analysis of 4 RCTs. Not a dual therapy comparison of interest (met+su v pio + su or su + met v pio + met).
Rajagopalan, 2006. American Journal of Geriatric Pharmacotherapy 4: 123-133.	Pooled post-hoc analysis of elderly patients from 4 RCTs. Not a dual therapy comparison of interest (met+su v pio + su or su + met v pio + met).
Ratner, 2011. 47th European Association for the Study of Diabetes (EASD) Annual Meeting.	Not a dual therapy comparison of interest. Patients were inadequately controlled on sulfonylurea ± metformin (only 16% of patients were on a SU alone).
Riddle, 2006. Diabetes/Metabolism	Child citation with no additional data (linked to Buse, 2004).

Citation Details	Exclusion reason
Research Reviews 22: 483-491.	
Salsali, 2012. Diabetes 61: A593-A594.	Child citation with no additional data (linked to Strojek, 2011).
Seino, 2010. Diabetologia 53: S329.	Abstract only (pre-2012 cut-off).
Seino, 2012. Journal of Diabetes Investigation 3: 517-525.	SU dose at randomisation (any dose allowed/ not half maximum dose).
Seino, 2012. Journal of Diabetes Investigation 3: 388-395	SU dose at randomisation (not stable dose for minimum of 4 weeks /not half maximum dose); Liraglutide dose lower than EU dose (0.6 mg, 0.9 mg).
Strojek, 2010. Diabetologia 53: S347-S348.	Abstract only (pre-2012 cut-off).
Tajima, 2011. Diabetology International 2: 32-44.	SU dose at randomisation (not half maximum dose). Sitagliptin dose not an EU licensed dose.

met, metformin; pio, pioglitazone; RCT, randomised-controlled trial; SE, standard error; SU, sulfonylurea
