# Supplementary material for: A systematic review and mixed-treatment comparison of dapagliflozin with existing anti-diabetes treatments for those with type 2 diabetes mellitus inadequately controlled by sulfonylurea monotherapy
Source: Diabetol Metab Syndr. 2014 Jun 11;6:73. doi: 10.1186/1758-5996-6-73 (PMC4085736; doi:10.1186/1758-5996-6-73)
Supplement: Additional file 3 — Key baseline characteristics for studies included in qualitative systematic review. [file 1758-5996-6-73-S3.pdf]

Table A.3. Key baseline characteristics for studies included in qualitative systematic review

Author, year	Treatment	N[a]	HbA1c, % [SD]	Mean duration of diabetes, years [SD]	Age, years [SD]	Male, n (%)	White/ Caucasian (excluding Hispanic), n (%)	Non-White / Caucasian, n (%)	BMI, kg/m^2 [SD]	FPG, mmol/L [SD] {mg/dl [SD]}
Buse, 2004[64]	placebo	123	8.7 [1.2]	5.7 [4.7]	55 [11]	77 (62.6%)	82 (66.7%)	41 (33.3%)	34 [5]	10.8 [3.2]
	exenatide 5 µg BID	125	8.5 [1.1]	6.3 [5.2]	55 [10]	74 (59.2%)	77 (61.6%)	48 (38.4%)	33 [6]	10.0 [2.5]
	exenatide 10 µg BID	129	8.6 [1.2]	6.6 [6.6]	56 [11]	74 (57.4%)	77 (59.7%)	52 (40.3%)	33 [6]	9.9 [2.8]
Garber, 2008[65]	placebo	144[b]	8.5 [1.0]	7.8 [5.8]	57.9 [10.5]	84 (58.3%)	97 (67.4%)	47 (32.6%)	31.0 [5.5]	10.3 [2.9]
	vildagliptin 50 mg QD	132[b]	8.5 [0.9]	6.9 [5.2]	58.6 [10.6]	78 (59.1%)	91 (68.9%)	41 (31.1%)	32.2 [4.9]	10.5 [3.0]
Hermansen, 2007[66]	placebo	106	8.43 [0.80]	8.0 [6.4]	55.2 [10.2]	58 (54.7%)	59 (55.7%)	47 (44.3%)	30.7 [6.4]	{184.9 [42.3]}
	sitagliptin 100 mg QD	106	8.42 [0.79]	7.2 [5.0]	54.4 [10.3]	56 (52.8%)	61 (57.5%)	45 (42.5%)	31.0 [6.7]	{182.6 [33.1]}
Lewin, 2012[67]	placebo	84	8.6 [0.7]	=<1 year: 7 (8.5%); 1 - 5 years: 29 (35.4%); >5 years: 46 (56.1%)	56.2 [10.2]	52 (61.9%)	34 (40.5%)	50 (59.5%)	28.2 [5.1]	{174.9 [49.0]}
	linagliptin 5 mg QD	161	8.6 [0.9]	=<1 year: 11 (7.0%); 1 - 5 years: 55 (34.8%); >5 years: 92 (58.2%)	57.2 [9.8]	77 (47.8%)	73 (45.3%)	88 (54.7%)	28.4 [5.0]	{182.0 [51.8]}

Author, year	Treatment	N[a]	HbA1c, % [SD]	Mean duration of diabetes, years [SD]	Age, years [SD]	Male, n (%)	White / Caucasian (excluding Hispanic), n (%)	Non-White / Caucasian, n (%)	BMI, kg/m^2 [SD]	FPG, mmol/L [SD] {mg/dl [SD]}
Strojek, 2011[34]	placebo	145[c]	8.15 [0.74]	7.4 [5.7]	60.3 [10.16]	71 (49.0%)	NR		BMI >= 25: 125 (86.2%); BMI >= 30: 66 (45.5%)	9.58 [2.07]
	dapagliflozin 10 mg QD	151[c]	8.07 [0.79]	7.2 [5.5]	58.9 [8.32]	66 (43.7%)			BMI >= 25: 120 (79.5%); BMI >= 30: 68 (45.0%)	9.55 [2.04]

[a]number of patients who had baseline values measured; [b]primary efficacy analysis population=at least one dose of study medication, baseline and at least one post-baseline HbA1c measurement; [c]full analysis set=randomised and at least one dose of study medication, baseline and at least one post-baseline efficacy measurement for one or more efficacy variable. Values are mean [SD] unless otherwise stated. BID, twice daily; BMI, body mass index; FPG, Fasting plasma glucose; QD, once daily; SD, standard deviation
