# Supplementary material for: A systematic review and mixed-treatment comparison of dapagliflozin with existing anti-diabetes treatments for those with type 2 diabetes mellitus inadequately controlled by sulfonylurea monotherapy
Source: Diabetol Metab Syndr. 2014 Jun 11;6:73. doi: 10.1186/1758-5996-6-73 (PMC4085736; doi:10.1186/1758-5996-6-73)
Supplement: Additional file 4 — Risk of bias summary graph. [file 1758-5996-6-73-S4.pdf]

	Buse, 2004	Garber, 2008	Hermansen, 2007	Lewin, 2012	Strojek, 2011
Selection bias: Random sequence generation	?	+	+	+	+
Selection bias: Allocation concealment	?	-	+	+	+
Performance bias: Blinding of participants and personnel	+	+	+	+	+
Detection bias: Blinding of outcome assessment	+	+	+	+	+
Attrition bias: Incomplete outcome data	-	+	+	+	+
Reporting bias: Selective reporting	+	+	+	+	+
Other sources of bias	+	+	+	+	+

Low risk of bias	+
Unclear risk of bias	?
High risk of bias	-
